# Recent Advances in Polymeric Membrane Integration for Organic Solvent Mixtures Separation: Mini-Review

**DOI:** 10.3390/membranes15110329

**Published:** 2025-10-30

**Authors:** Abdellah Halloub, Wojciech Kujawski

**Affiliations:** 1 Faculty of Chemistry, Nicolaus Copernicus University in Toruń, 7 Gagarina Street, 87-100 Torun, Poland; 2 Chemistry Department, Universidade NOVA de Lisboa, 2829-516 Caparica, Portugal

**Keywords:** membrane separation, organic-organic separation, pervaporation, nanofiltration, organic solvent nanofiltration, reverse osmosis

## Abstract

Membrane technology offers considerable potential for enhancing or partially replacing conventional separation techniques, which could eventually lead to substantial energy savings. This review focuses on recent advancements in membrane separation technologies including organic solvent pervaporation (OSPV), organic solvent reverse osmosis (OSRO), organic solvent nanofiltration (OSN), and organic solvent ultrafiltration (OSUF) that are increasingly vital in the pharmaceutical, biochemical, and petrochemical industries. Although hybrid and inorganic membranes exhibit promising performance, polymeric membranes provide advantages in scalability and processability. The development of materials capable of operating under demanding conditions that include exposure to organic solvents, high temperatures, extreme pH levels, and oxidative environments remains critical. Here, we examine recent innovations in membrane materials and their integration into organic solvent systems. Key challenges, including material swelling, fouling, and scaling, are discussed, along with recent strategies to address these issues. Finally, we identify emerging research directions that could drive further progress in membrane technology for organic media applications.

## 1. Introduction

Separation processes are widely applied technologies in the chemical and pharmaceutical industries, enabling the efficient purification of raw materials and reducing impurity levels to meet industry standards [1]. These industries rely heavily on organic solvents for reactions and separations [2], making the recovery and recycling of these solvents essential for environmental sustainability and industry longevity. Traditionally, thermal separation processes have been the primary means of organic solvent recovery, yet they account for approximately 10–15% of global energy consumption [3], which conflicts with the shift from a linear economy towards a circular one.

In response, membrane separation has emerged as an economically sustainable alternative to thermal processes, offering energy consumption that is about one-tenth that of traditional thermal methods [4]. Membrane technology, which has been developing rapidly since the 1960s, is gaining attention for its high energy efficiency and potential to transform industrial processes [5]. Various techniques, including organic solvent nanofiltration (OSN), organic solvent reverse osmosis (OSRO), organic solvent forward osmosis (OSFO), organic solvent ultrafiltration (OSU), and pervaporation (PV), are now applied in a wide range of applications such as solvent recovery, solute separation, and purification applications across several industries.

Despite recent advancements in polymer materials and membrane fabrication, designing organic solvent-resistant membranes with high performance remains challenging. Indeed, effective membranes must be stable across a range of organic solvents, withstand exposure to acids and bases, offer high selectivity, and maintain permeation rates. High-stability polymers, such as polyimide (PI) [6], polymers of intrinsic microporosity (PIM) [7], polybenzimidazole (PBI) [8], and polyetheretherketone (PEEK) [9], have greatly enhanced membrane resilience in harsh environments. Besides that, additional post-treatment modifications further improve these membranes solvent resistance [10,11].

While several reviews have summarized membrane separation in organic media and advances in polymeric membranes [12,13,14], this article focuses on recent progress specifically from the perspective of membrane materials. It begins with an overview of advancements in polymer-based membranes and then examines the performance stability challenges in organic media, analyzing the causes and potential solutions proposed by researchers.

## 2. Application of Membrane Separation in Industries

Membranes are used in several industries, including drug separation and purification, the petrochemical sector, and beyond. This section highlights recent advances in the application of membranes in organic media.

### 2.1. Oleochemical Industry

The oleochemical industry is a key component of the bio-based economy, centered on the production of renewable chemicals obtained from natural fats and oils. Oleochemicals, in contrast to petrochemicals, are derived from plant oils such as palm, soybean, and coconut, as well as from animal fats, and in some cases are synthesized through microbial metabolism [15]. These compounds, which include fatty acids, alcohols, esters, and glycerol, are widely used in applications such as personal care, food processing, lubricants, and biodegradable materials [16]. Traditional separation methods like distillation and solvent extraction are often energy-intensive and generate significant waste [17]. In this context, membrane-based technologies, particularly OSN, are gaining attention for their ability to recover oils, recycle solvents, and extract high-value compounds with improved energy efficiency and selectivity [18].

Membrane-based separation has emerged as a promising approach for sustainable and efficient processing in the oleochemical industry, particularly for oil recovery, solvent recycling, and the extraction of value-added compounds. Shi et al. [19] investigated the separation of triglycerides and free fatty acids from oil mixtures using a range of commercial Evonik membranes. Under static conditions, the Duramem 500 membrane exhibited a sharp decline in permeance from 1.02 to 0.06 L m^−2^ h^−1^ bar^−1^ as oil concentration increased, with only a slight drop in glyceryl trilinoleate rejection from 86% to 84.8% over a feed range of 5 to 50 wt.%. This loss in performance was attributed to the high viscosity and surface fouling by oil components. However, under cross-flow operation using a 20 wt.% oil-acetone feed, the membrane achieved significantly improved performance, with permeance values three to six times higher than in static mode, highlighting the operational benefits of dynamic filtration. Addressing the need for membranes with enhanced solvent stability, Li et al. [20] developed composite membranes based on trifluoropropylmethylsiloxane dimethylsiloxane (PDMS-PTFPMS) and polyvinylidene fluoride (PVDF), combining the high hexane permeability of PDMS with the chemical resistance of PTFPMS. The F50-M membrane demonstrated over 95% oil rejection and stable hexane permeability of 3.06 kg m^−2^ h^−1^ bar^−1^ over 32 days, with performance attributed to its fluorine-rich microstructure and surface properties. These findings indicate strong potential for hexane recovery in oleochemical solvent extraction processes. Similarly, an AF2400/PTFE composite membrane was applied by Li et al. [21] for hexane separation from soybean oil-hexane mixtures. The membrane maintained high oil rejection above 98% and stable permeate flux between 0.8 and 1.1 L m^−2^ h^−1^ bar^−1^, even after 1440 h of aging in pure hexane, confirming its suitability for long-term operation in harsh solvent environments. Extending OSN to the recovery of minor compounds, Ghazali et al. [22] evaluated polyimide-based DuraMem and PuraMem membranes for the direct separation of carotenes from palm oil. PuraMem 280 achieved the best selectivity, raising carotene concentration in the permeate from 0.60 to 0.79 g/L using hexane, whereas other membranes showed poor discrimination between carotene and triglycerides. The study also employed a coupled solution-diffusion and film theory model to describe carotene transport, underlining OSN’s potential as a viable alternative to traditional extraction methods for recovering high-value oleochemicals from vegetable oils.

### 2.2. Pharmaceutical and Biotechnology Industry

Membrane separation, especially microfiltration, is essential in the pharmaceutical industry, particularly for producing injectable drugs. Strict regulations require standardized production processes to ensure product safety and sterility [23]. Microfiltration removes particles and, crucially, all bacteria, typically using filters rated at 0.22 µm. A filter is considered sterile if it achieves a log reduction factor of over 7, meaning that it can remove all bacteria even with an initial concentration of 10 million bacteria per square centimeter. For added safety, two filters are often used in series to ensure complete bacteria and virus removal. Biopharmaceutical production generally involves between 10 and 20 sequential membrane-based separation stages [24]. Membrane chromatography modules, or membrane adsorbers, are widely used, offering purification through affinity, ion exchange, or hydrophobic interactions. NatriFlo^®^ membrane chromatography, for example, employs a polyacrylamide hydrogel supported by a fiber matrix, with a 0.4 µm effective pore size that enables high surface adsorption and rapid flow driven by convection. These systems allow high flow rates with low pressure, making them ideal for disposable setups, with required volumes achieved by stacking multiple membranes or pleated cartridges. In bioprocessing, membranes are primarily used in flow-through chromatography, where impurities bind to the membrane while the target product flows through unimpeded. Many manufacturers, including Sartorius (Sartobind, Göttingen, Germany), Pall (Mustang, NY, USA), MilliporeSigma (NatriFlo, Hong Kong, China), and Cytiva (FibroSelect, Marlborough, MA, USA), offer anion exchange membranes with quaternary amine functionality, which effectively bind DNA and remove endotoxins at capacities above 20 mg/mL [25]. In studies, NatriFlo^®^ HD-Q (MilliporeSigma, Hong Kong, China) demonstrated approximately 5-log removal of viruses such as mouse minute virus (MMV) and murine leukemia virus (MLV) from monoclonal antibody products, highlighting these membrane adsorbers as effective for viral clearance in purifying products derived from mammalian cells [26].

Moreover, OSN is gaining increasing attention in pharmaceutical applications, particularly for drug synthesis and intermediate purification. OSN enables the separation of active pharmaceutical ingredients (APIs) and fine chemicals in solvent-rich environments, offering a low-energy and thermally gentle alternative to traditional methods such as distillation. The use of polyimide-based thin-film composites, inorganic–organic hybrids, and ionic liquid-assisted layers in OSN membrane fabrication enhances their suitability for recovering high-value compounds and removing impurities during complex, multi-step synthesis. Although OSN is not yet as standardized as microfiltration in bioprocessing, it shows strong potential for integration into pharmaceutical manufacturing pipelines, especially when handling temperature-sensitive or solvent-soluble compounds [27].

### 2.3. Chemical and Petrochemical Industry

Membrane technology plays a vital role in liquid-phase separations within the petrochemical industry, offering energy-saving alternatives to conventional methods like distillation and solvent extraction. One major application is in PV, used for separating close-boiling or azeotropic mixtures such as aromatic-aliphatic blends, which are difficult to separate by traditional techniques. Thomas et al. [28] developed a mixed matrix membrane (MMM) by incorporating multi-walled carbon nanotubes (MWCNTs) into a natural rubber (NR) and nitrile butadiene rubber (NBR) blend. Designed specifically for separating a 50:50 benzene/cyclohexane mixture, the resulting nanocomposite membranes demonstrated a total flux of 1.265 kg m^−2^ h^−1^ and a separation factor of 1.59 at ambient temperature. Notably, the membranes exhibited a clear preference for the aromatic component, indicating effective aromatic selectivity during the pervaporation process. Xi et al. [29] proposed ionic liquid copolymerized waterborne polyurethane (IL-co-PU) membranes for the same separation task. Among these, the membrane with 10 wt% ionic liquid (PU-10) showed the best performance, achieving a high permeation flux of 310 g m^−2^ h^−1^ and a separation factor of 8.3 during pervaporation of a 50/50 wt% benzene/cyclohexane mixture at 50 °C. These values represent significant improvements of 150% in flux and 240% in separation factor compared to the pristine polyurethane membrane (PU-0). In addition, PU-10 exhibited one of the highest reported pervaporation separation index (PSI) values for polymer membranes, reaching 2263 g m^−2^ h^−1^. Similarly, Zahlan et al. [30] investigated poly(ethylene-co-vinyl alcohol) (E-VOH) and its carbon nanotube-enhanced version (E-VOH/CNT) for the same application. Their findings revealed that the incorporation of CNTs significantly expanded the absorption area, increasing the flux to 740 g m^−2^ h^−1^, while the separation factor reached 9.03 and the pervaporation separation index climbed to 5942.2 g m^−2^ h^−1^ over a 3 h process for the azeotropic mixture. In contrast, the unmodified E-VOH membrane exhibited a lower flux of 280 g m^−2^ h^−1^, a higher separation factor of 12.90, and a PSI of 3332 g m^−2^ h^−1^ under the same conditions. Comparative performance analysis showed that the E-VOH/CNT membrane achieved 2.64 times the flux of the plain E-VOH membrane, although its separation factor was reduced to 70% of the unfilled version. These studies collectively highlight the effectiveness of combining nanomaterials, ionic liquids, and tailored polymer matrices to optimize both permeability and selectivity in pervaporation processes.

Membranes are also employed in solvent dewaxing, where OSN systems recover solvents like methyl ethyl ketone and toluene from dewaxed oil, significantly reducing energy consumption associated with vacuum distillation. Namvar-Mahboub et al. [31] developed a thin-film nanocomposite (TFN) membrane incorporating amino-functionalized UZM-5 nanoparticles into a polyamide selective layer, specifically targeting the recovery of MEK and toluene. The optimized membrane, containing 0.02 *w*/*v*% UZM-5, achieved a permeate flux of 13.85 L m^−2^ h^−1^ and an oil rejection of 96.27% at 15 bar, with a MEK/toluene mass ratio of 0.81. The decreased mass ratio compared to the neat TFC membrane indicated an enhanced interaction between toluene and the zeolite surface, suggesting that surface affinity played a more dominant role than molecular sieving at this composition. In a separate study, Monjezi et al. [32] proposed a chemically crosslinked polyaniline (PANi) membrane supported on woven polyester and stabilized via glutaraldehyde treatment to improve solvent resistance. Their PANi membrane demonstrated a permeate flux of ~10 L m^−2^ h^−1^ and an oil rejection of approximately 69% under 35 bar. They further analyzed the impact of operational parameters, noting that while increasing pressure and feed flow improved performance, higher oil concentrations diminished flux due to concentration polarization and increased viscosity. Compared to the TFN system, the PANi membrane offers a simpler fabrication route and cost-effectiveness, albeit with reduced separation efficiency.

### 2.4. Organic Compounds Separation Using Hansen Solubility Parameters

The prediction of solvent-membrane interactions is an essential aspect of designing and selecting membranes for organic solvent separations. Among the different modeling methods, the Hansen solubility parameter (HSP) approach has been extensively used due to its simplicity and strong correlation with experimental permeability and selectivity data [33]. HSP theory is based on the concept that the total cohesive energy density (δ) of a material can be divided into three components: dispersion (δ_d_), polar (δ_p_), and hydrogen-bonding (δ_h_) interactions [34]. These parameters collectively describe the molecular interactions that govern solubility and sorption processes in polymers and solvents.

The compatibility between a polymer membrane and a solvent can be quantified by the Hansen distance (Ra), expressed as:

(1) $$R_{a}=\sqrt{4{\left(\delta_{d1}-\delta_{d2}\right)}^{2}+{\left(\delta_{p1}-\delta_{p2}\right)}^{2}+{\left(\delta_{h1}-\delta_{h2}\right)}^{2}}$$

where subscripts 1 and 2 correspond to the solvent and polymer, respectively. A smaller *R*a value indicates higher affinity and greater solubility of the solvent in the polymer. In the context of membrane separation, this implies that solvents with HSP values closer to those of the membrane material tend to show higher sorption and, consequently, higher permeability. Conversely, solvents with larger HSP distances exhibit lower sorption and are therefore more effectively rejected [35].

Each polymer occupies a characteristic region in “Hansen space,” defined by a center point (δd, δp, δh) and a radius of interaction R_0_. Solvents lying inside this sphere (R_a_ < R_0_) are thermodynamically compatible and will typically cause swelling or dissolution, while those outside the sphere (R_a_ > R_0_) show poor compatibility and are less likely to penetrate or plasticize the polymer.

The ratio of these two quantities is known as the Relative Energy Difference (RED):

(2) $$RED=\frac{R_{a}}{R_{0}}\;$$

A RED < 1 implies good affinity (potential swelling or dissolution), whereas RED > 1 denotes poor affinity and generally ensures membrane stability against the solvent [36]. Thus, assessing both *R*_*a*_ and *R*_0_ enables prediction of whether a solvent will merely wet the membrane surface, cause controlled swelling beneficial for permeation, or lead to polymer damage.

Several studies have demonstrated the usefulness of this criterion for evaluating membrane–solvent compatibility. Huang et al. used a “HSP-guided design” strategy for selecting compatible polymer-filler combinations in mixed-matrix membranes (MMMs). They calculated the HSPs of candidate polymers and nanoscale fillers, then used the “like seeks like” principle to ensure that the fillers HSPs were close to those of the polymer matrix (i.e., small *R*_*a*_). This close match reduced interfacial defects, improved compatibility, and resulted in MMMs with enhanced mechanical integrity and separation performance. Their results showed that MMMs designed through HSP matching achieved better retention of flux and selectivity compared to mismatched combinations, thereby validating that HSP-based screening is effective for preselecting filler/polymer pairs [37]. Jun et al. proposed a quantitative model linking variation in HSP distances to changes in the effective mass-transfer pathways within membranes. The authors demonstrated that as the HSP distance Ra between solvent and membrane increases, the degree of swelling or perturbation in the membrane matrix decreases, narrowing the mass-transfer channels and reducing permeability. Conversely, when Ra is small (i.e., high affinity), swelling can widen transport pathways, increase flux but possibly compromise selectivity. This model provided a direct functional relationship between HSP difference and effective diffusivity in the membrane, thereby showing how HSP metrics can predict how solvent-membrane affinity affects transport behavior [38]. Ewah et al. used HSP-based calculations (specifically the RED) to predict which polymeric membrane materials would remain stable in a hydrophobic deep eutectic solvent (DES) mixture. They exposed different membranes (polysulfone, cellulose acetate, PVDF, PBI) to the DES for extended periods and observed dissolution or structural degradation for those with RED < 1 (i.e., the solvent was within or close to the polymer’s solubility sphere). Indeed, polysulfone (RED = 0.6) and cellulose acetate (RED = 0.9) dissolved fully within 24 h, while PVDF (RED = 1.9) and PBI (RED = 1.1) remained intact during a 7-day exposure. This strong correlation between RED > 1 and membrane structural stability provides a compelling experimental validation of the HSP/RED criterion as a predictor for whether a solvent will dissolve (or overly swell) a polymer [39]. Kv et al. investigates whether the HSP distance Ra between post-treatment solvent solutions (e.g., DMF, acetonitrile, ethanol) and the polyamide active layer correlates with changes in permeability (PWP) and salt rejection of thin-film composite (TFC) membranes. The authors found that although differences in *Ra* provided insight into how “aggressive” a solvent might be toward the polyamide, HSP alone was not fully predictive of the observed performance changes for all membranes. For example, acetonitrile (with *R*_*a*_ = 8.3) caused large increases in water permeability for some NF membranes but negligible effect on RO membranes. They discuss possible reasons: (i) the composite structure (support + polyamide) complicates interactions, (ii) swelling or structural changes are not fully captured by HSP, and (iii) deviations from ideal behavior or interfacial phenomena. Nonetheless, the authors conclude that HSP distance remains a useful (though not complete) descriptor for solvent-membrane affinity and can be part of a broader predictive toolkit [40].

## 3. Membrane Separation

### 3.1. Organic Solvent Pervaporation (OSPV)

**Figure 1:**
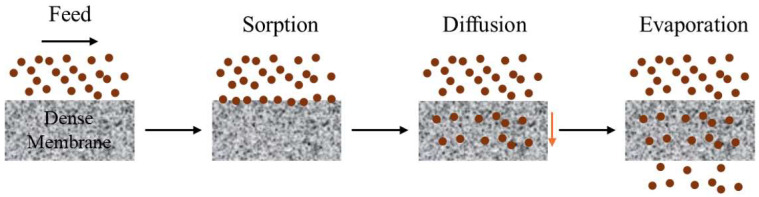
Solution-diffusion mechanism in the dense membrane.

PV is recognized for its capacity to substantially decrease the energy and resource usage of manufacturers in acquiring high-purity components through the utilization of automated and easily scalable equipment [5]. It proves to be an effective method for separating azeotropic and closely boiling mixtures owing to its reliance on the solution-diffusion mechanism, which is not liable upon the relative volatility of chemicals but rather on differences in the sorption and diffusion characteristics of the feed solution, along with membrane characteristics [41]. In this process, constituents of a liquid feed mixture permeate through a membrane, evaporating on the opposite side to generate a permeate (Figure 1). The mass transport across the membrane is driven by the chemical potential difference. This driving force is generated by using either a vacuum pump or an inert purge on the permeate side to keep the vapor pressure of the permeate lower than the partial pressure of the feed liquid [41]. Mass transfer in dense polymer membranes is commonly accounted for by the solution-diffusion model [42]. PV separates substances by essentially separating their vapors using a dense polymer membrane. The process operates through a mechanism that involves three key stages. First, the feed molecules are absorbed onto the upstream side of the membrane. Next, the absorbed components move through the membrane by diffusion. Finally, components are desorbed at the downstream membrane surface, effectively achieving the separation. Simultaneously, the sorption and diffusion steps may complement or oppose each other, depending on the transport characteristics of PV membranes [43].

**Figure 2:**
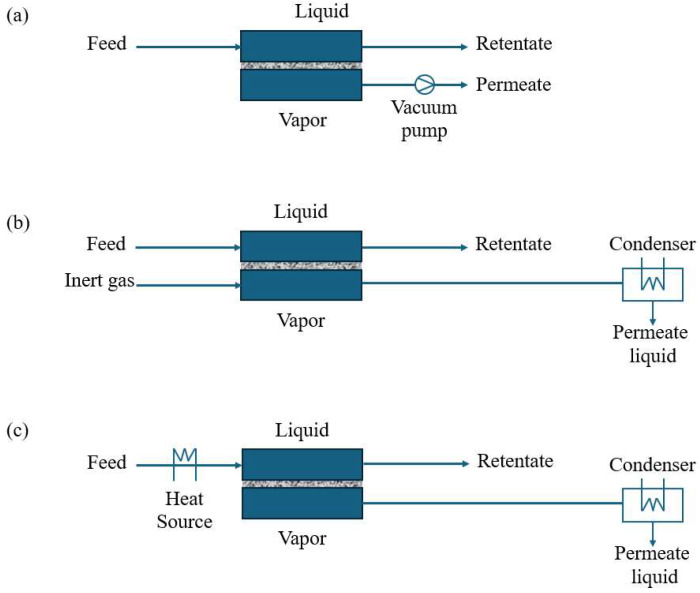
Schematics of potential PV process: (**a**) vacuum PV, (**b**) sweep-gas PV, (**c**) and thermo-PV.

In PV, there are three important module configurations, which are vacuum PV, sweep-gas PV, and thermo-PV, that mainly differ in how the permeate side is managed to maintain a strong partial pressure difference (Figure 2) [44,45]. In vacuum PV, a vacuum pump lowers the pressure on the permeate side so that, once molecules pass through the membrane, they immediately enter a low-pressure environment, where they can be collected or condensed. In sweep-gas PV, an inert gas (commonly nitrogen) flows along the permeate side, continuously removing the permeated vapor and thus maintaining a low partial pressure [44]. Finally, thermo-PV involves controlling temperatures (for instance, heating the membrane or providing different thermal conditions on the permeate side) to enhance the driving force for mass transfer; by creating a favorable temperature gradient, it both increases flux and may improve separation efficiency.

The performance of a membrane in the PV process is characterized by the total flux and separation factor. These factors are often utilized to assess and compare the performance of various PV membranes. Total flux (J) (kg m^−2^ h^−1^) is calculated by measuring the mass of permeate (m) generated over a specific time (t) relative to the effective membrane surface (A) and expressed by Equation (1) [44]:

(3) $$J=\frac{\Delta m}{A\times \Delta t}\;$$

The separation factor in PV is a measure of the effectiveness of a membrane in separating two components of a mixture. It quantifies how well one component permeates through the membrane relative to another component. The separation factor (β_A/B_) between two components A and B in PV is defined as:

(4) βAB=yAyBxAxB

$y_{A}$ and $y_{B}$ are the mole fractions of components A and B in the permeate (vapor phase after passing through the membrane). $x_{A}$ and $x_{B}$ are the mole fractions of components A and B in the feed mixture. If βAB>1, the membrane selectively favors the permeation of component A over component B, resulting in an enrichment of A in the permeate compared to the feed. A higher separation factor indicates better separation efficiency. If βAB=1, the membrane does not prefer either component, meaning there is no selective separation between A and B.

Although interest in synthesizing polymer membranes for PV dates back nearly a century, only in recent years have significant advancements been achieved, as demonstrated by a series of recent comparative studies using a range of membrane materials and modification strategies. Knozowska et al. [46] conducted two notable studies using heterogeneous polymer-inorganic composite membranes, each aimed at different separation targets. In the first study, they focused on membranes made from poly(ether block amide) and poly(dimethylsiloxane), modified with alumina oxides grafted with various silane agents: octyltriethoxysilane (C6), 1H,1H,2H,2H-perfluorooctyltriethoxysilane (FC6), tetradecyltriethoxysilane (C12), and 1H,1H,2H,2H-perfluorotetradecyltriethoxysilane (FC12). Their membranes were evaluated for separating ethyl acetate/ethanol and ethyl acetate/isopropanol mixtures, showing that the incorporation of grafted alumina oxide significantly improved separation performance. Notably, membranes grafted with C6 and FC12 delivered the highest separation efficiencies, with separation factors of β_EtAc/EtOH_ = 3.9 and β_EtAc/IPA_ = 3.4 for C6, and β_EtAc/EtOH_ = 4.3 and β_EtAc/IPA_ = 3.6 for FC12. Furthermore, the FC12-modified Al_2_O_3_ membranes demonstrated the highest hydrophobicity, as indicated by a water contact angle of 115° ± 0.1°. In their second study, Knozowska et al. [47] applied a different modification strategy, fabricating PVA composite membranes embedded with NaY zeolite on a polyamide-6 (PA6) support. These were designed for organic-organic separations, particularly ethanol and ethyl tert-butyl ether (EtOH/ETBE) mixtures. Performance was evaluated through the separation factor and thickness-normalized PV separation index (PSI_N_). The PVA-NaY/PA6 membrane achieved a separation factor of 2.3 and a PSI_N_ of 288 μm g m^−2^ h^−1^, representing improvements of 143% and 160%, respectively, over the unmodified PVA/PA6 membrane. Additionally, the water contact angle improved from 55.6° to 75.2°, indicating enhanced hydrophobicity and stability. While Knozowska’s approaches involved polymer-inorganic hybrids focused on moderate selectivity improvements across alcohols and esters, Dong et al. [48] adopted a different approach by developing ultrathin organosilica membranes derived from Bis(triethoxysilyl)acetylene (BTESA). These membranes were used to separate a 10 wt% methanol and 90 wt% dimethyl carbonate (DMC) mixture, which is an azeotrope particularly challenging to separate. Their membranes showed exceptional selectivity, with a separation factor of ~120 and permeation fluxes of 2–4 kg m^−2^ h^−1^ at 50 °C. The superior performance was attributed to the membrane chemical stability and preferential absorption of methanol enabled by molecular size exclusion. Taking a different approach, Banerjee et al. [49] created composite membranes using coated sodium montmorillonite (NaMMT) clay, embedded within a PVA matrix. The clay was coated with an acrylonitrile and acrylic acid copolymer (5:1 molar ratio) using in situ intercalative emulsion polymerization, with the coated clay comprising 8% of the membrane weight. Their system was tested for benzene–cyclohexane mixtures, and the coated clay significantly enhanced benzene sorption, leading to very high separation factors. Depending on benzene content in the feed, the membranes reached fluxes of 65.38, 85.34, and 168.56 g m^−2^ h^−1^, with separation factors of 187.96, 172.72, and 137.16, respectively, for feeds containing 0.5, 8, and 50 wt.% benzene. Post-PV, the benzene content was enriched up to 99.2 wt.%, showing strong sorption-driven selectivity. Finally, Zhang et al. [50] employed a nanochannel engineering strategy by synthesizing a COF-LZU8 covalent organic framework functionalized with thioether-containing hydrazone linkages and silver ions, which was incorporated into a commercial Pebax 2533 membrane. This system was tailored for aromatic/aliphatic separations, specifically toluene/n-heptane mixtures. The presence of functionalized COFs enabled the formation of selective nanochannels, significantly improving membrane performance compared to unmodified Pebax. The resulting membrane achieved a flux of 293 g m^−2^ h^−1^ and a separation factor of 4.03 for a 50 wt.% toluene/n-heptane mixture.

In contrast to other organic solvent membrane processes, the OSPV technique is unique in that the permeate is obtained in vapor form. This introduces an additional evaporation selectivity component that acts in conjunction with the intrinsic sorption-diffusion selectivity of the membrane [51]. Therefore, OSPV simultaneously depends on both the membrane physicochemical affinity toward the feed components and the volatility differences between the permeating species.

### 3.2. Organic Solvent Reverse Osmosis (OSRO)

**Figure 3:**
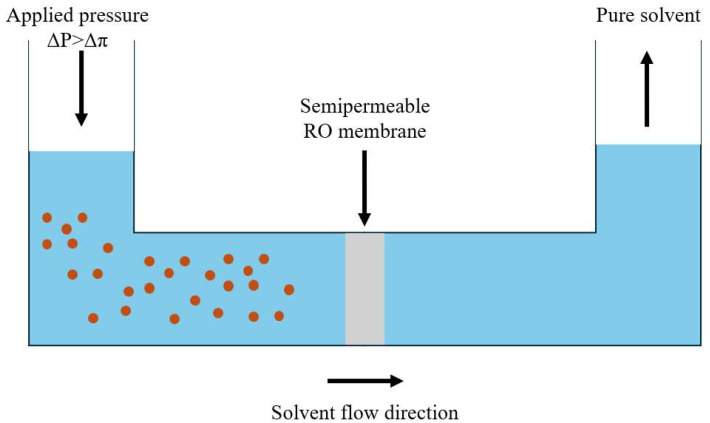
Reverse osmosis process.

In natural osmosis, a solvent moves from areas of low solute concentration to regions with higher concentration, aiming to equalize solute levels across a membrane, which creates osmotic pressure. Applying pressure to membrane reverse the flow. When the applied pressure exceeds the osmotic pressure (Δ*p* > Δπ), the solvent passes through the membrane, leaving solute behind and producing purified solvent (Figure 3). The effectiveness of the OSRO membrane depends on two main factors: flux and rejection [52].

(5) J=A(∆p−∆π)

*J* represents the rate of solvent flow, ∆*p* represents the difference in transmembrane pressure (TMP), ∆*π* signifies the disparity in osmotic pressure between the feed side and the permeate side, and A represents an intrinsic solvent permeability coefficient characterizing the membrane [52].

(6) $$R=\left(1-\frac{C_{p}}{C_{f}}\right)\times 100\%$$

R represents the solute rejection percentage, C_p_ is the solute concentration in the permeate, and C_f_ solute concentration in the feed.

OSRO is used not just for separating solutes but also for separating mixtures of organic solvents. This technology is particularly promising for separating organic liquid mixtures, primarily due to its high energy efficiency. It mainly works through a solution-diffusion mechanism, where solvent molecules dissolve in the membrane and then diffuse through it to the permeate side [53]. The separation of organic liquids depends largely on their different sorption or diffusion behaviors within the membranes. However, since OSRO is still in the early stages of development, the separation mechanism needs more research and validation [54]. As mentioned earlier, the OSRO process relies solely on a chemical difference driving force, meaning that even small defects in the membrane can cause pressure-driven fragments, which reduces separation efficiency and makes OSRO very sensitive to flaws. Additionally, OSRO requires relatively high operating pressures to overcome high osmotic pressures and maximize productivity [55]. The osmotic pressure can vary with different organic mixtures, so the pressure must be adjusted for specific conditions. As the molecular sizes of the components in the mixture are similar, size separation becomes less effective, and the separation increasingly relies on entropy or polarity selectivity. Consequently, membrane design strategies can vary significantly across different separation systems. OSRO membranes need to maintain high stability in organic media and withstand high pressures in order to provide excellent separation performance. These demanding requirements contribute to the slower development of OSRO membranes compared to the rapid advancements seen in OSN research.

Over the last few years, numerous advancements have been made in the design of OSRO membranes, with researchers exploring diverse materials and fabrication techniques to enhance performance in solvent separation. One notable approach was demonstrated by Kushida et al., who developed a hydrophobic thin-film composite (TFC) membrane by incorporating fluorine-based chemistry into the design. Using 5-trifluoro-1,3-phenylenediamine (TFMPD) in the aqueous phase and trimesoyl chloride (TMC) in the organic phase, they synthesized a membrane capable of selectively permeating nonpolar solvents such as aliphatic and aromatic hydrocarbons, which traditional hydrophilic polyamide membranes (e.g., those made with m-phenylenediamine, MPD) could not. Their membrane achieved selective permeation of toluene and a 93.9% rejection rate for 1,3,5-triisopropylbenzene (TIPB), indicating excellent separation performance in hydrocarbon-rich systems [56]. Guan et al. took a surface chemistry-driven route by modifying polyketone (PK) membranes for OSRO. They used a nonwoven polyester fabric and glass plate to support a PK layer formed via phase separation in a methanol-water coagulation bath. After solvent exchange and drying, the membrane surface was treated with a 2 wt% aqueous MPD solution at 80 °C, followed by interfacial polymerization with TMC to form a polyamide layer. This method emphasized the impact of surface modification on membrane morphology and performance. As a result, their membranes exhibited enhanced solvent resistance and increased permeance, highlighting how controlling the formation of crumpled polyamide layers can lead to improved OSRO separation efficiency [57]. Adopting a hybrid inorganic–organic material strategy, Kitamura et al. [58] developed TiO_2_-ZrO_2_-OCL composite membranes using three different organic chelating ligands (OCLs): acetylacetone (ACA), methyl-2,4-heptanedione (ACAiB), and 2,6-dimethyl-3,5-heptanedione (ACA2iP). These membranes were tested for methanol selectivity in 90 wt% methanol/10 wt% toluene mixtures under 5 MPa transmembrane pressure. They reported total fluxes between 0.072 and 0.968 kg m^−2^ h^−1^ and separation factors from 3.2 to 180, depending on the ligand used. Notably, the M-ACAiB membrane delivered high separation efficiency but low permeance, while the M-ACA2iP membrane showed higher permeance with lower selectivity, demonstrating the trade-off between these performance metrics based on ligand structure. Pursuing ultrathin fluoropolymer membranes, Chau and Sirkar [59] investigated a 1.67 μm -thick film made from a PDD-TFE copolymer (perfluoro-2,2-dimethyl-1,3-dioxole and tetrafluoroethylene), supported on expanded PTFE (e-PTFE). This membrane was tested for separating various binary organic solvent mixtures. For systems like NMP-ethanol and DMSO-ethanol with 90–95 wt% ethanol, the permeate was found to be pure ethanol, indicating strong selectivity. However, at lower ethanol concentrations (5–10 wt%), the permeate matched the feed composition, indicating no separation. Similar behavior was observed in methanol-DMSO and methanol-NMP mixtures. For toluene/methanol systems, selective toluene permeation was achieved only at ≥90 wt% toluene, while no selectivity was observed in heptane-dodecane/ethanol mixtures. These findings underscore the membrane’s preference for polar protic solvents over aprotic solvents at high concentrations and highlight its limitations in nonpolar systems. Focusing on spin-coated membranes for nonpolar–polar separation, Liu et al. [60] coated Teflon AF2400 onto a solvent-resistant polyketone support and evaluated the membrane in ethanol/1-hexane binary mixtures. Operating at 4 MPa, the membrane achieved a separation factor of 9.5 for 1-hexane over ethanol, along with a flux of 1.6 kg m^−2^ h^−1^ (or permeance of 0.04 kg m^−2^ h^−1^ bar^−1^). The high selectivity for nonpolar 1-hexane was attributed to the nonpolar nature of AF2400, which favors nonpolar solvent permeation while resisting polar solvents, which effectively complements the membrane selectivity trends observed by Chau and Sirkar. Building further on polyketone support systems, Liu et al. [61] also employed interfacial polymerization to develop an additional OSRO membrane for organic liquid mixtures. This membrane exhibited robust stability and achieved remarkable separation factors of 8.4 (toluene), 11.1 (pentane), 14.9 (hexane), and 38.0 (heptane) from 10 wt% mixtures in methanol under 3 MPa, with consistent fluxes around 5 L m^−2^ h^−1^. These values not only demonstrate strong selectivity for large nonpolar molecules over smaller polar ones but also position this membrane ahead of conventional reverse osmosis and OSN membranes in terms of both stability and separation performance.

In fact, both OSRO and OSPV rely fundamentally on the solution-diffusion mechanism, where permeating molecules dissolve in the membrane and subsequently diffuse across it. However, their driving forces differ substantially: OSPV is driven by a chemical-differential generated by vacuum or sweep-gas operation, whereas OSRO uses a transmembrane pressure gradient that exceeds the osmotic pressure of the feed mixture. As a result, OSPV is generally more suitable for separating azeotropic or volatile component mixtures, where phase change enhances selectivity, while OSRO performs better for liquid–liquid separations involving components with comparable volatilities but differing polarities or molecular sizes [62]. In practice, OSPV achieves higher selectivity at lower throughputs, whereas OSRO offers higher fluxes but requires elevated pressures and more mechanically robust membranes.

### 3.3. Organic Solvent Nanofiltration (OSN)

OSN, also known as solvent-resistant nanofiltration (SRNF), is a membrane-based technology that uses pressure to separate small molecules in organic solvents [63]. This process typically operates at pressures between 5 and 30 bar, depending on the specific solvent and solute properties, with temperature adjustments based on the solvent boiling point and membrane stability. OSN membranes generally possess pores sized between 0.5 to 2 nm, enabling separation based on molecular size or weight [64]. One major benefit of OSN is its effectiveness in separating temperature-sensitive molecules, where traditional methods often struggle owing to high temperatures that can damage sensitive compounds. Unlike these thermal methods, OSN works at lower temperatures, relying on pressure to achieve separation, making it ideal for industries needing to preserve the structure and function of sensitive molecules [65]. OSN allows selective separation and concentration of valuable compounds without the risk of thermal degradation, making it efficient. The selectivity of an OSN membrane is often described by its Molecular Weight Cut-Off (MWCO), which indicates the membrane retention capacity [66]. MWCO represents the molecular weight of a solute for which the membrane retains 90% of the molecules. Generally, OSN membranes are suited for separating molecules in the range of 100 to 2000 g/mol [66].

The permeate flux in OSN can be expressed using Equation (5) [66]:

(7) $$J_{P}=L_{p}*\Delta p$$

where $J_{P}$ is the permeate flux (L h^−1^ m^−2^), $L_{p}\;$ is the membrane’s permeability (L h^−1^ m^−2^ bar^−1^), and Δ*p* is the transmembrane pressure (bar). The Δ*p* is the pressure difference across the membrane, driving the solvent through the membrane, as described in Equation (6):

(8) $$TMP=\frac{P_{feed}+P_{retentate}}{2}-P_{permeate}$$

$L_{p}$ represents the solvent permeability under a given pressure and can be described by Equation (7) [67]:

(9) $$L_{p}=\frac{1}{{\eta *R}_{m}}$$

η is the viscosity of permeate (Pa s), which is always a function of the temperature. The $R_{m}$ is membrane resistance (m^−1^).

With ongoing advancements in processing techniques, OSN is steadily evolving into a more sustainable and eco-friendly separation technology. A key determinant of OSN efficiency lies in the careful design and selection of membranes tailored to specific separation challenges. Recent studies illustrate a variety of innovative approaches to membrane fabrication, each optimizing performance through unique material combinations and structural modifications. Alduraiei et al. [68] introduced a rapid and practical method for fabricating covalent organic polymer (COP)-based composite membranes through interfacial polymerization completed in under 10 s. Fabrication was carried out directly on a polyacrylonitrile substrate, eliminating the need for additional transfer steps. To enhance hydrophobicity, fluorine-rich groups were incorporated into the polymer network. These membranes demonstrated impressive OSN performance, achieving a toluene permeance of 11 L m^−2^ h^−1^ bar^−1^ and over 95% rejection of Congo red (687 g/mol), making the method both time-efficient and effective for nonpolar solvent separations. Expanding on the idea of rapid fabrication but with a focus on green chemistry, Zheng et al. [69] developed an ionic liquid (IL)-assisted approach for preparing polyamide/poly(m-phenylene isophthalamide) (PMIA) thin-film composite membranes. They employed the eco-friendly IL [EMIm][OAc] to prepare PMIA substrates with controlled pore sizes (7–11 nm), and used another IL, [BMIm][Tf_2_N], as the organic phase for interfacial polymerization with piperazine, polyethyleneimine, and aromatic acyl chlorides. This dual-IL system slowed monomer diffusion, resulting in a thinner selective layer and thus significantly enhanced permeance, from 0.6 to 2.15 L m^−2^ h^−1^ bar^−1^, while maintaining >99% rejection for both vitamin B12 and Congo red. The study highlighted how viscosity control through ILs can be leveraged to fine-tune membrane performance. Focusing on structural optimization, Su et al. [70] fabricated a polyimide hollow fiber (HF) substrate with an outer skin layer, which was then functionalized through interfacial polymerization. The process employed low-concentration MPD, TMC, and additives like sodium dodecyl sulfate (SDS) and triethylamine (TEA) to form a thin-film composite OSN membrane with an average surface roughness below 3 nm. This membrane achieved nearly 100% rejection of rhodamine B (RDB) and an ethanol permeance exceeding 12 L m^−2^ h^−1^ MPa^−1^. Subsequent coating with graphene oxide (GO) further increased ethanol permeance to ~20 L m^−2^ h^−1^ MPa^−1^, without compromising rejection, showing how nanomaterial layering can boost both flux and selectivity. In a related approach, Hong et al. [71] pursued a thin-film nanocomposite (TFNi) strategy by adding interlayers to PMIA substrates, followed by interfacial polymerization in an IL medium. They synthesized five distinct transition metal-piperazine complexes (Co-PIP, Ni-PIP, Mn-PIP, Zn-PIP, Cu-PIP) and found that the PMIA/Co-PIP/PA membrane offered the best performance, achieving an ethanol permeance of 2.93 L m^−2^ h^−1^ bar^−1^ and 95% rejection of solutes > 697 Da. This approach underlines how metal–organic coordination chemistry in interlayers can regulate membrane interface formation and selectivity. Further exploring polymer chemistry, Zhang et al. [72] synthesized polyimide membranes for OSN by polymerizing 4,4′-(hexafluoro-isopropylidene) diphthalic anhydride with 1,4-bis(4-aminophenoxy)benzene diamine, then casting the membrane on glass and precipitating it in a non-solvent phase. These membranes were tested for dye separation in methanol, achieving 94.6% rejection of Rose Bengal, 94.2% for methyl blue, 63.2% for Victoria blue B, and 58.2% for crystal violet, with stable operation over 60 h. This work showcased the robustness and chemical resistance of polyimide membranes in aggressive organic environments, albeit with somewhat lower rejection for smaller solutes.

### 3.4. Organic Solvent Ultrafiltration (OSUF)

Organic solvent ultrafiltration (OSUF) is a pressure-driven separation process that uses porous membranes that are stable in organic solvents. It is primarily used to filter out larger organic molecules from these solvents [14]. OSUF typically employs membranes with a molecular weight cut-off (MWCO) of around 1000 to 100,000 Da, making the process effective for separating large molecules or suspended solids without much risk of pore clogging [73]. It should be remembered that OSN has a much lower MWCO range of about 100 to 2000 Da, making it suitable for finer separations, especially to purify active pharmaceutical ingredients or organic acids. OSU membranes have a porous structure, with pore sizes between 10 to 100 nm, and the permeate flux (J) through the membrane can be calculated using Darcy’s law ((8), (9)) [74,75]:

(10) $$J=\frac{∆P}{µ\times R}$$

Or

(11) $$J=\frac{L\times ∆P}{µ}$$

Here, ∆P is the transmembrane pressure difference, R indicates the membrane’s resistance, L represents membrane permeability, and μ corresponds to the viscosity of the fluid traversing the membrane pores. While nanofiltration membranes offer superior purification efficiency, ultrafiltration requires less energy and achieves higher permeate flux. The membrane material also determines which organic solvents can be used, leading to distinct applications for organic-solvent nanofiltration (OSN) and organic-solvent ultrafiltration (OSUF) [76]. OSN is predominantly employed in the petrochemical, food, bulk and fine chemical, and pharmaceutical sectors due to its ability to separate molecules with precise selectivity under solvent-rich conditions. In comparison, OSUF is more commonly applied in the food industry, particularly for recovering valuable compounds from industrial waste and for use in the edible oil sector, with increasing attention toward its integration into biorefinery processes.

Among recent advancements in OSUF membrane technology, Liu et al. [6] introduced a novel PI/PEI@TiO_2_ ultrafiltration membrane to improve the performance limitations of traditional polyimide (PI) membranes, which often struggle with polar solvent compatibility and low flux. Their fabrication strategy combined non-solvent-induced phase separation, chemical crosslinking, and interfacial in situ biomineralization. The incorporation of polyethyleneimine (PEI) facilitated the formation of TiO_2_ nanoparticles, creating additional transport channels while simultaneously crosslinking with the PI matrix to improve chemical resistance. The resulting PI/PEI@TiO_2_-5 membrane (with 5 wt% TiO_2_) demonstrated exceptional stability across a range of organic solvents, including strong polar aprotic, polar, and nonpolar types, and achieved a notably high flux exceeding 50 L m^−2^ h^−1^ bar^−1^ in DMF, marking a substantial enhancement over unmodified PI membranes. Focusing on tuning selectivity rather than structural reinforcement, Oxley et al. [77] explored the optimization of polybenzimidazole (PBI) membranes through grafting modification. They manipulated the ratio of two grafting agents: a high-molecular-weight polymer modifier (Elastamine RE-1-1000, 1000 g/mol, Huntsman, Duxford (UK)) and a low-molecular-weight compound (2-methoxyethylamine, 75 g/mol). Membranes modified solely with the long-chain grafting agent exhibited a molecular weight cut-off (MWCO) of 2000 g/mol in acetonitrile. However, by increasing the concentration of the small-molecule modifier, they were able to cap unreacted grafting sites, enabling precise control of the membrane’s MWCO in the range of 2000 to 20,000 g/mol in acetonitrile. This demonstrated a practical method for fine-tuning pore size and selectivity without compromising solvent stability.

Abdellah et al. investigated the efficiency of ultrafiltration (UF) for the degumming of crude canola oil using bio-derived solvents as a sustainable alternative to conventional hexane extraction. They prepared a miscella of canola oil with terpene-based solvents such as d-limonene and p-cymene, and applied a 5 kDa ultrafiltration membrane to separate phospholipids and other impurities from the oil phase. The results showed phospholipid rejections higher than 95%, while maintaining a stable flux throughout the operation period, indicating that the membrane performed reliably in a fully organic environment. Moreover, the composition of the permeate confirmed that triglycerides were retained almost completely, preserving the nutritional and physicochemical quality of the refined oil. These results demonstrated that ultrafiltration can be successfully integrated into solvent-based refining systems using renewable, low-toxicity solvents, providing a water-free and environmentally friendly alternative to conventional degumming methods [78]. Aryanti et al. investigated the use of a polyethersulfone (PES) ultrafiltration membrane for the degumming of crude palm oil (CPO) dissolved in isopropanol, aiming to develop a cleaner and solvent-based alternative to conventional aqueous degumming. The study examined the influence of oil concentration and temperature on the permeate flux, phospholipid rejection, and fouling behavior during ultrafiltration. The results showed that increasing oil concentration in the feed mixture caused a noticeable decline in permeate flux due to higher viscosity and greater deposition of colloidal impurities on the membrane surface. An increase in temperature from 30 °C to 40 °C initially enhanced flux as a result of reduced viscosity, but a further rise to 45 °C led to flux reduction, possibly because of membrane compaction or swelling in the organic solvent. The PES membrane demonstrated high selectivity toward phospholipids, achieving over 99% rejection at 30% oil concentration and approximately 93% at 40%, confirming the membrane effectiveness in removing polar impurities from the oil-isopropanol miscella. In contrast, the separation of free fatty acids (FFAs) was negligible, since these small, nonpolar molecules readily permeated through the membrane [79].

To better illustrate the diversity in membrane fabrication and performance in organic phase, Table 1 summarizes selected recent studies, including materials, methods, applications, and observed limitations.

In general, each organic solvent membrane process presents specific advantages and limitations. OSPV provides excellent selectivity for azeotropic or closely boiling systems but can suffer from low permeate flux, vacuum energy demand, and potential membrane swelling in aggressive solvents. OSRO enables pressure-driven liquid separations with high recovery but faces challenges such as high operating pressures, membrane compaction, and sensitivity to structural defects. OSN and OSUF, while mature in concept, continue to struggle with the trade-off between flux and selectivity and the long-term chemical stability of polymer matrices in strong solvents. Despite these challenges, recent efforts toward hybrid organic–inorganic membranes, covalent organic frameworks (COFs), and fluorinated or ionic-liquid-modified polymers have significantly improved solvent resistance and permeability.

## 4. Challenges and Limitations

### 4.1. Swelling

Swelling in polymeric membranes occurs when the material absorbs solvent molecules, which leads to the expansion of material. This effect is especially noticeable in organic solvents, where the polymer chains tend to interact easily with nonpolar or weakly polar solvents. These interactions increase the space between the polymer chains, causing the membrane to swell. Swelling affects membrane performance by raising permeability, which can improve flux but often reduces selectivity, as the larger spaces in the polymer network allow more unwanted molecules to pass through [86]. Excessive swelling can also damage the membrane’s structure, causing deformation, weakening, or even rupture under pressure. Swelling is a particular challenge in applications like OSN and pervaporation (OSPV), where high selectivity is crucial. Therefore, managing swelling is essential for enhancing membrane durability and effectiveness in these applications [87].

To address the persistent challenge of membrane swelling without compromising separation performance, researchers have developed several innovative strategies aimed at improving membrane stability under harsh operating conditions. One approach was proposed by Liu et al. [88], who engineered a composite “sandwich” structure by adding a polypropylene (PP) layer to a dual-layer polydimethylsiloxane/polyamide (PDMS/PA) membrane, forming a PP/PDMS/PA configuration. In this design, the PP layer acts as a protective barrier, shielding the underlying PDMS separation layer from direct exposure to fermentation broth and mechanical stress, while the PDMS layer maintains its role as the selective separation layer and bridges the PP and PA layers. The polyamide (PA) layer, in turn, serves as the structural support base. This multilayer architecture significantly reduced swelling in water, ethanol, and fermentation broth by 21%, 6%, and 17%, respectively, compared to the original PDMS/PA membrane. Taking a different strategy focused on internal molecular design, Xu et al. [89] designed a thin-film composite membrane featuring an internal cross-linked network suitable for OSN. The membrane was fabricated by reacting 4,4′-bipyridine with brominated poly(2,6-dimethyl-1,4-phenylene oxide) (BPPO). The resulting high degree of crosslinking restricted polymer chain mobility, effectively limiting membrane swelling to just 2.01%. This design highlights the power of chemical crosslinking in controlling dimensional stability in solvent environments. Further advancing material robustness through molecular design, Su et al. [90] developed three types of conjugated microporous polymer (CMP) membranes using a two-step polymerization process. Constructed on a rigid aromatic backbone characterized by strong carbon-carbon (C-C) bonds, these CMP membranes offered exceptionally high surface areas and precisely tuned pore sizes. As a result, they exhibited very high permeability, sharp selectivity for OSN applications, and excellent anti-swelling properties. Importantly, their permeability and rejection performance remained virtually unchanged even after prolonged solvent exposure, emphasizing the effectiveness of using rigid conjugated structures for dimensional stability.

### 4.2. Fouling

Membrane fouling happens when unwanted particles build up on the membrane surface or clog its pores, reducing permeability and overall performance. Fouling is influenced by factors such as the quality of the feed solution and membrane properties [91]. This accumulation of fouling materials is a major obstacle to the widespread use of membranes in liquid separation. Indeed, fouling decreases permeate flow and system efficiency, which in turn requires more frequent cleaning and raises operational costs [92].

Preventing membrane fouling remains essential for advancing and broadening the adoption of membrane technologies. To mitigate fouling risks in feed solutions and enhance membrane efficiency and operational lifespan, researchers have developed a range of strategies. These approaches include pretreating feed solutions, optimizing operational conditions, designing new fouling-resistant materials and structures, selecting effective cleaning methods, and often combining multiple tactics for comprehensive fouling control. Among these, the development of antifouling membranes stands out as a particularly critical method for long-term performance improvement [92]. In pursuit of this goal, Gu et al. [93] developed a novel polymeric OSN membrane featuring an asymmetric structure based on fluorinated polymeric networks (FPNs). The FPNs were synthesized from m-phenylenediamine (MPD), dopamine (DA), and 1H,1H,2H,2H-perfluorodecane-thiol (PFDT) via Michael addition and Schiff-base reactions. Their FPN-modified thin-film composite (FPN-TFC) membranes mimicked biological structures by presenting a looser, hydrophilic front surface and a denser, hydrophobic rear surface. This biomimetic design led to an impressive ethanol permeance of 15.7 L m^−2^ h^−1^ MPa^−1^ and a high rejection rate of 99.5% for Rose Bengal (RB), tested under 1.5 MPa and 25 °C using 0.05 g L^−1^ RB ethanol solution. The FPN-TFC membranes outperformed traditional TFC membranes by nearly sixfold and demonstrated excellent antifouling properties, suggesting strong potential for future OSN applications. Building on the idea of enhancing surface properties for antifouling, Zhou et al. [94] adopted a bio-inspired approach by chemically modifying lignin for use in nanofiltration (NF) membrane interfacial polymerization. Their lignin-based modification (LG-NH_2_) significantly improved membrane hydrophilicity, effectively reducing both reversible and irreversible fouling. Additionally, LG-NH_2_ membranes optimized pore size and improved permeability, while maintaining good selectivity; only a slight reduction in Na_2_SO_4_ rejection and a minor decline in MgCl_2_ rejection were observed. This work demonstrated that lignin modification can successfully balance antifouling performance, permeability, and selectivity. In further advancement toward structural antifouling enhancements, Wei et al. [95] introduced a method for fabricating thin-film nanocomposite (TFNi) membranes by incorporating a ZIF-8 metal–organic framework (MOF) interlayer using a one-step phase inversion and crosslinking process. This method involved polyimide (PI) precipitation, crosslinking PI with hexanediamine, and the simultaneous formation of ZIF-8 nanocrystals via interfacial diffusion. The resulting membranes, specifically the OSFOISG-2.5 variant, achieved a molecular weight cut-off of ~500 Da, exhibited high permeances for deionized water (9.73 L m^−2^ h^−1^ bar^−1^) and tetrahydrofuran (THF) (1.62 L m^−2^ h^−1^ bar^−1^) in reverse osmosis mode, and increased ethanol fluxes in forward osmosis (2.10 ± 0.11 L m^−2^ h^−1^) and pressure-retarded osmosis (2.97 ± 0.31 L m^−2^ h^−1^). Furthermore, a 10 h DMF activation treatment enhanced the membrane’s solvent permeance without negatively affecting polystyrene rejection during reverse osmosis, confirming the durability and adaptability of the structure.

### 4.3. Scaling

Membrane systems face persistent challenges due to the presence of ions such as Ca^2+^, Mg^2+^, and SO_4_^2-^ in the feed solution. These ions tend to accumulate on membrane surfaces or within pores, leading to mineral scaling through retention effects [96]. Over time, scaling can clog membranes, decrease their performance, increase osmotic pressure, and potentially cause irreversible damage [97]. To stop scaling, current industrial strategies primarily rely on chemical agents or scale inhibitors added to the feed solution. While effective, these chemical treatments pose economic and environmental concerns, indicating the urgent need for innovative membrane designs with intrinsic anti-scaling properties. One such approach was developed by Shen et al. [98] who introduced a technique combining in situ interfacial polymerization with a citric acid post-treatment to minimize scaling. Their membrane, tested for desalination applications, showed only a 33.5% drop in water permeance during recovery, compared to a 65.8% drop in untreated membranes, thus demonstrating substantial improvement in resisting mineral fouling. Focusing on surface modification to enhance smoothness and reduce scaling sites, Peng et al. [99] employed very low concentrations of COF monomers, 1,3,5-triformylphloroglucinol (Tp) and benzidine (Bd), to form a uniform covalent organic framework (COF) interlayer on a polyimide substrate. This interlayer was followed by a polyamide (PA) formation through interfacial polymerization of trimesoyl chloride (TMC) and low-concentration m-phenylenediamine (MPD), with subsequent crosslinking and activation steps. The resulting interlayered thin-film composite (i-TFC) membrane achieved an exceptionally smooth surface, enhancing anti-scaling properties, while maintaining high performance with 99.5% rejection of Rose Bengal (RB), a DMF permeance of 103.0 L m^−2^ h^−1^ MPa^−1^ (one of the highest reported for DMF), and an ethanol permeance of 65 L m^−2^ h^−1^ MPa^−1^.

### 4.4. Stability

Because most organic-organic separation processes take place under harsh chemical and thermal conditions, a key challenge in membrane development is achieving strong chemical and thermal stability [100]. Among current options, polymeric membranes, particularly those made from polyimides with rigid molecular chains, have shown promise due to their solvent resistance, scalability, and structural durability. These properties make them suitable for PV membranes used in separating complex mixtures such as toluene and iso-octane [101]. However, polyimide membranes are still tend to swell in pure organic solvents, and enhancing the permeation flux remains a critical area for improvement, particularly through the refinement of the polyimide selective layer. In comparison, zeolite membranes offer superior separation performance and stability due to their uniform, rigid crystalline pores, which can be tuned in size and surface affinity to suit specific separation tasks [102]. These structural advantages make zeolites particularly effective in harsh environments. Nevertheless, the industrial-scale production of defect-free zeolite membranes remains a significant obstacle due to the complexity of hydrothermal synthesis techniques [103]. Among zeolites, hydrophobic MFI-type membranes have demonstrated excellent efficient separation of xylene isomers [104]. Further enhancements in their performance have been achieved using techniques such as oriented growth and nanosheet seeding to control membrane morphology and reduce defects.

## 5. Conclusions and Perspectives

Membrane separation technologies have evolved significantly, becoming crucial in pharmaceuticals, biotechnology, and petrochemical applications. From microfiltration to nanofiltration and reverse osmosis, membranes offer efficient and sustainable solutions for separating and purifying substances at the molecular and ionic levels. However, challenges such as membrane fouling, selectivity, and permeability persist, prompting ongoing research into innovative strategies for surface modification and membrane material design. The development of advanced materials such as mixed-matrix membranes (MMMs) and thin-film composite (TFC) membranes holds great promise for achieving superior separation performance, particularly in OSN and OSPV. By incorporating nanomaterials and optimizing membrane structures, researchers aim to overcome limitations and pave the way for the commercialization of highly selective and permeable membranes for organic solvent separations.

However, further research and development efforts are needed to translate these innovations into practical applications and address the evolving challenges in various fields. To meet future application needs, polymeric membranes should be designed with several essential features: they must resist a wide range of solvents, both polar and nonpolar, while offering high selectivity to distinguish between molecules of similar molecular weights. Additionally, these membranes should achieve high rejection rates for smaller solute particles while maintaining enhanced solvent permeance, which would help reduce the required membrane surface area. These advancements will be important for optimizing membrane performance and efficiency in organic solvent separation.

## Figures and Tables

**Table 1:** Comparative summary of recent membrane separation in organic medium: materials, fabrication methods, applications, and performance.

Membrane Type	Material	Fabrication Method	Application	Conditions	Performance	Limitations	Ref.
OSN	polyamide	Interfacial polymerization of Polyethyleneimine (PEI), piperazine (PIP) and trimesoyl chloride (TMC)	Rose Bengal and acid fuchsin	0.2 MPa	116.0 and 45.0 L m^−2^ h^−1^ MPa^−1^ permeability in acetone and isopropanol. 99.9% and 91.8% rejection to rose bengal (1017 Da) and acid fuchsin (585 Da).	DMF still swelling	[80]
OSPV	Polyether	CE (3,4-Epoxycyclohexylmethyl) and DOX (3-ethyl-3-[(3-ethyloxetan-3-yl)methoxymethyl]oxetane) cationic photopolymerization	Separation of ethanol/n-hexane and ethanol/cyclohexane	25 °C, vacuum pump (1.3 kPa)	Separation factor of up to 162.81 for ethanol/n-hexane azeotrope and 41.89 for ethanol/cyclohexane azeotrope	Not specified	[81]
OSPV	carboxyl-functionalized polyimide	Solvent-casting	Separation alcohol/nonpolar solvent	40 °C, 0–8·10^−4^ MPa	For methanol/toluene flux was 0.13 kg m^−2^ h^−1^ and Separation factors was 140. For methanol/methyl tert-butyl ether flux was 0.09 kg m^−2^ h^−1^ and separation factors was 3065 For ethanol/cyclohexane flux was 0.11 kg m^−2^ h^−1^ and separation factors was 406.	Not specified	[82]
OSRO	Polyketone supported polyamide	Interfacial polymerization	Separation alcohol/nonpolar solvent	3–5 MPa, 25–60 °C	Separation factor: 45 MeOH from MeOH/Toluene mixture, separation factor: 210.0 MeOH form MeOH/Hexane, Flux: Around 2.1–2.4 kg m^−2^ h^−1^	Gradual Decline in Selectivity over time due to PA chain relaxation	[83]
OSRO	perfluoro-2,2-dimethyl-1,3-dioxole and tetrafluoroethylene (Teflon^®^ AF2400 from Sigma-Aldrich Co., Burlington, USA) onto a polyketone support	Spin-coating	Toluene/1,3,5-Triisopropylbenzene separation	25 °C, 4.0 MPa	96.2% 1,3,5-Triisopropylbenzene rejection, 0.7 L m^−2^ h^−1^ MPa^−1^ permeance	Limited commercial maturity	[60]
OSN	3-amino-1-adamantanol (AAMO) with acyl chloride	interfacial polymerization	Fast green (FCF) in ethanol	0.3 MPa, 25 °C	295.0 L m^−2^ h^−1^ MPa^−1^ for pure MeOH and FCF/MeOH rejection of 84.4%	Not specified	[84]
OSUF	polyimide/polyethyleneimine@TiO2	coupling the non-solvent-induced phase transformation (NIPs), chemical crosslinking and interfacial in situ biomineralization	Polyethylene glycol rejection from N,N-dimethylformamide (DMF)	0.4 MPa, 25 °C	654.0 L m^−2^ h^−1^ MPa^−1^	Not specified	[6]
OSN	Polydopamine with Polyamide on PTFE nanofibrous substrate	Electrospinning of PTFE/FEP/PEO, PDA coating, interfacial polymerization of PIP-TMC	Rose Bengal (RB) rejection in EtOH and DMF	0.2 MPa, 25 °C	In EtOH: 59.5 L m^−2^ h^−1^ MPa^−1^ flux, 96.3% RB rejection; In DMF: 23.2 L m^−2^ h^−1^ MPa^−1^, 92.2% RB rejection	The membrane exhibited a relatively lower permeance in DMF	[85]
